# Gene-diet interactions with polymorphisms of the *MGLL* gene on plasma low-density lipoprotein cholesterol and size following an omega-3 polyunsaturated fatty acid supplementation: a clinical trial

**DOI:** 10.1186/1476-511X-13-86

**Published:** 2014-05-24

**Authors:** Catherine Ouellette, Iwona Rudkowska, Simone Lemieux, Benoit Lamarche, Patrick Couture, Marie-Claude Vohl

**Affiliations:** 1 Institute of Nutrition and Functional Foods (INAF), Laval University, 2440 Hochelaga Blvd., Quebec, QC, Canada; 2 CHU de Québec Research Center – Endocrinology and Nephrology, Quebec, QC, Canada

**Keywords:** Omega-3 polyunsaturated fatty acids, Nutrigenomics, *MGLL*, LDL cholesterol, LDL particle size

## Abstract

**Background:**

Omega-3 (n-3) polyunsaturated fatty acid (PUFA) consumption increases low-density lipoprotein (LDL) cholesterol (C) concentrations and particle size. Studies showed that individuals with large, buoyant LDL particles have decreased risk of cardiovascular diseases. However, a large inter-individual variability is observed in LDL particle size. Genetic factors may explain the variability of LDL-C concentrations and particle size after an n-3 PUFA supplementation. The monoglyceride lipase (MGLL) enzyme, encoded by the *MGLL* gene, plays an important role in lipid metabolism, especially lipoprotein metabolism. The aim of this study was to investigate if polymorphisms (SNPs) of the *MGLL* gene influence the variability of LDL-C and LDL particle size in response to an n-3 PUFA supplementation.

**Methods:**

210 subjects completed the study. They consumed 5 g/d of a fish oil supplement (1.9-2.2 g eicosapentaenoic acid and 1.1 g docosaexaenoic acid) during 6 weeks. Plasma lipids were measured before and after the supplementation period and 18 SNPs of the *MGLL* gene, covering 100% of common genetic variations (minor allele frequency ≥0.05), have been genotyped using TaqMan technology (Life Technologies Inc., Burlington, ON, CA).

**Results:**

Following the n-3 PUFA supplementation, 55% of subjects increased their LDL-C levels. In a model including the supplementation, genotype and supplementation*genotype effects, gene-diet interaction effects on LDL-C concentrations (rs782440, rs6776142, rs555183, rs6780384, rs6787155 and rs1466571) and LDL particle size (rs9877819 and rs13076593) were observed for the *MGLL* gene SNPs (p < 0.05).

**Conclusion:**

SNPs within the *MGLL* gene may modulate plasma LDL-C levels and particle size following an n-3 PUFA supplementation. This trial was registered at clinicaltrials.gov as NCT01343342.

## Background

Omega-3 (n-3) polyunsaturated fatty acids (PUFA) have been studied for many years in relation to cardiovascular diseases (CVD). It has been demonstrated that eicosapentaenoic acid (EPA, 20:5n3) and docosahexaenoic acid (DHA, 22:6n3) consumption from marine sources may be beneficial in reducing CVD risk and mortality [1-4]. This association may be related in part to the plasma triglyceride (TG) lowering effect of n-3 PUFA [5-7]. However, less is known about the low-density lipoprotein (LDL) cholesterol (C) increase following an n-3 PUFA supplementation that has also been observed in several studies [8-10]. Elevated LDL-C levels are a major CVD risk factor [11]. Consequently, reducing LDL-C is a main therapeutic target in CVD prevention programs, as recommended by the National Cholesterol Education Program Adult Treatment Panel III (NCEP-ATPIII) [12]. However, the cholesterol content of LDL particles is not the sole issue; LDL particle size may also influence CVD risk. Actually, large and buoyant particles are less atherogenic than small and dense particles [13-15]. It has been proposed that the increase in LDL-C levels caused by an n-3 PUFA supplementation may be associated with a shift in the particle distribution from small, dense to large and buoyant [16-18]. Yet, the results in the literature are inconsistent. Some studies have found a favourable shift in the LDL-particle size distribution [17,19-22], while some others observed an equally distributed elevation of small and large LDL particles following an n-3 PUFA supplementation [23-25].

The large inter-individual variability of the metabolic response to an n-3 PUFA supplementation between individuals may be caused by genetic factors [26,27]. Single nucleotide polymorphisms (SNPs) have previously been associated with the variability of plasma lipid response to an n-3 PUFA intake [28,29].

The monoglyceride lipase (MGLL) enzyme works together with the hormone-sensitive lipase to hydrolyze intracellular TG stores in adipocytes, and other cells, to fatty acids and glycerol. MGLL may also complement lipoprotein lipase in completing hydrolysis of monoglycerides resulting from the degradation of lipoprotein TG [30]. MGLL is also the main contributor to 2-arachidonoylclycerol (2-AG) degradation, a central component of the endocannabinoid signaling system [31]. MGLL is encoded by the *MGLL* gene, which is located in chromosome 3. To our knowledge, this gene has not been studied in relation to blood lipids or n-3 PUFA intakes yet.

The aim of this study was to test whether single nucleotide polymorphisms (SNPs) of a gene involved in TG metabolism could contribute to the n-3 PUFA supplementation effect on LDL-C levels and particle size.

## Methods

### Subjects

254 participants were recruited from September 2009 to December 2011 from the Quebec City metropolitan area via advertisements in local newspapers and electronic messages sent to university students and employees. Subjects had to be aged between 18 and 50 years old, be non-smokers and free of any thyroid or metabolic disorders requiring treatment such as diabetes, hypertension, severe dyslipidemia and coronary heart disease. Their body mass index (BMI) had to be between 25 and 40 kg/m^2^. Participants could not have taken n-3 PUFA supplements 6 months before the beginning of the study. A total of 210 subjects completed the n-3 PUFA supplementation period and 208 subjects had LDL-C and particle size data available for further analyses. The ethics committees of Laval University Hospital Research Center and Laval University approved this experimental protocol. This trial was registered at clinicaltrials.gov as NCT01343342.

### Study design and diets

Subjects enrolled in the study completed a 2-week run-in period in which they received individual dietary instructions by a trained registered dietitian based on *Eating Well with Canada’s Food Guide* recommendations. They were asked to follow these recommendations and to maintain their body weight stable throughout the protocol. To ensure stable nutrient intakes, instructions were given regarding the n-3 PUFA dietary intake: subjects could not exceed two fish or seafood servings per week, had to prefer white flesh fishes instead of fatty fishes (examples were given) and avoid enriched n-3 PUFA food such as milks, juices, breads and eggs. They were not allowed to consume n-3 PUFA supplements (such as flaxseed), vitamins or natural health products during the study. Also, participants had to limit their alcohol intake during the study to two drinks per week.

After the 2-week run-in period, each subject received a bottle containing needed n-3 PUFA capsules for the following 6 weeks. Then, they were instructed to take five (1 g fish oil each) capsules per day (Ocean Nutrition, Nova Scotia, Canada), providing a total of 3–3.3 g of n-3 PUFA (1.9-2.2 g EPA and 1.1 g DHA) per day. Counting the remaining capsules assessed compliance, as they were provided in sufficient quantity for 6 weeks. Participants were asked to report any deviation during the protocol, their alcohol and fish consumption as well as side effects if they occurred. Before each phase, subjects received detailed written and verbal instructions on their diet.

A registered dietitian administered a validated food-frequency questionnaire (FFQ) to each participant before the run-in period [32]. This FFQ was based on typical food items available in Quebec and contained 92 items; 27 items had between 1 and 3 subquestions. The subjects were asked how often they consumed each item per day, per week, per month, or none at all during the month prior. Many examples of portion sizes were provided to estimate precisely the portion consumed. Moreover, participants were instructed on how to complete a 3-day (two weekdays and one weekend day) food journal pre- and post-n-3 PUFA supplementation. Dietary intakes were analysed using Nutrition Data System for Research software version 2011 (Nutrition Coordinating Center, University of Minnesota, Minneapolis, MN).

### Anthropometric measurements

Body weight, height and waist circumference were measured according to the procedures recommended by the Airlie Conference [33] and were taken before the run-in period as well as pre- and post- n-3 supplementation. BMI was calculated as weight in kilograms per height in square meter (kg/m^2^).

### Biochemical parameters

Blood samples were collected from an antecubital vein into vacutainer tubes containing EDTA after 12 hours overnight fast and 48 hours alcohol abstinence. Blood samples were drawn before the run-in period to identify and exclude participants with metabolic disorders. Afterwards, selected participants had blood samples taken before and after the n-3 PUFA supplementation period. Plasma was separated by centrifugation (2500 x g for 10 min at 4°C) and samples were aliquoted and frozen for subsequent analyses. Plasma total-cholesterol (TC) and TG concentrations were measured using enzymatic assays [34,35]. The high-density lipoprotein cholesterol (HDL-C) fraction was obtained after precipitation of very low-density lipoprotein and LDL particles in the infranatant with heparin manganese chloride [36]. LDL-C was calculated with the Friedewald formula [37]. Apolipoprotein (Apo) B-100 concentrations were measured in plasma by the rocket immunoelectrophoretic method of Laurell [38]. As previously described [15,39], LDL particle size was obtained by non-denaturing 2–16% polyacrylamide gel electrophoresis of whole plasma. LDL size was extrapolated from the relative migration of plasma standards of known diameter [40]. The estimated diameter for the major peak within each densitometric scan was used to identify LDL particle size. Analysis of pooled plasma standards showed that LDL peak measurements and mean particle diameters were highly reproducible (interassay coefficients of variation of <2%).

### SNP selection and genotyping

**Figure 1:**
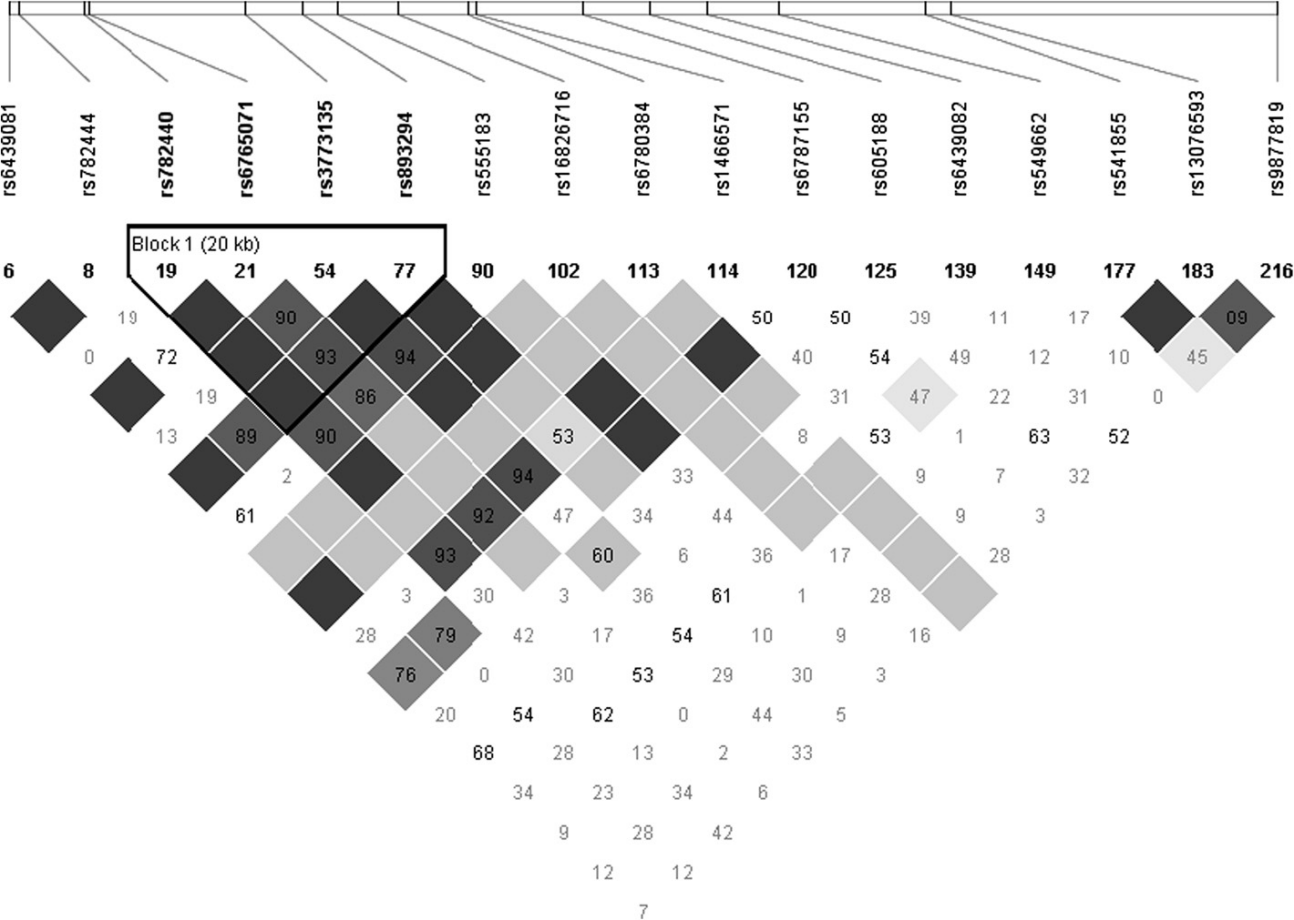
**Linkage disequilibrium (LD) plot of selected SNPs in the *MGLL *** **gene.**

SNPs in *MGLL* were identified with the International HapMap project SNP database (data release 28, Phase II + III), based on the National Center for Biotechnology Information (NCBI assembly B26, dbSNP build 126). The Utah residents with ancestry from northern and western Europe (CEU) population was used. Data were retrieved and pairwise linkage disequilibrium (LD; R^2^ values) between SNPs was computed using the Haploview software V4.2. The tagger selection algorithm was used to determine tag SNPs from SNPs with a minor allele frequency (MAF) > 5% (R^2^ cut-off ≥ 0.8). Afterwards, we examined LD between 18 SNPs in *MGLL* covering all common variations in this gene, using the LD Plot procedure in Haploview V4.2. A minimum of 85% of the most common SNPs had to be captured by tag SNPs. For *MGLL,* 18 SNPs were sufficient to cover the entire area, with 99% of the most common SNPs included (Figure 1). The SIGMA GenElute Gel Extraction Kit (Sigma-Aldrich Co., St. Louis, MO, USA) has been used to extract genomic DNA. DNA was mixed with TaqMan Universal PCR Master Mix (Life Technologies Inc.), with a gene-specific primer and with probe mixture (pre-developed TaqMan SNP Genotyping Assays; Life Technologies Inc.) in a final volume of 10 μL. Then, genotypes were determined using a 7500 RT-PCR System and analysed using ABI Prism SDS version 2.0.5 (Life Technologies Inc.). Minor allele homozygotes with a genotype frequency < 5% were grouped with heterozygotes for statistical analyses.

### Statistical analysis

Statistical analyses were performed with SAS statistical software V9.2 (SAS Institute, Cary, NC, USA). Subjects with missing values for calculated LDL-C and for LDL particle size were excluded from statistical analysis. The ALLELE procedure was used to verify the departure from Hardy-Weinberg equilibrium (HWE) and to calculate MAF. Values that were not normally distributed were log_10_-transformed before analyses. ANOVAs were used to test for significant differences for metabolic and anthropometric characteristics as well as to test for differences between various nutrient intakes before and after the n-3 PUFA supplementation. The MIXED procedure for repeated measures was used to test for the effects of the genotype, the supplementation and the genotype*supplementation interaction on LDL-C and LDL particle size in a model including age, sex and BMI. Further adjustments for protein as well as carbohydrate intakes (at baseline or changes during the intervention) were also performed. Subjects were divided in two groups on the basis of their LDL-C levels variation between pre- and post-supplementation (delta LDL). Participants who increased their LDL-C levels (≥0% variation) were assigned to the positive responders group for LDL-C while those who decreased their LDL-C levels (<0% variation) were in the negative responders group for LDL-C. The same was done for LDL particle size variation. Frequencies of positive responders and negative responders in the different genotypic groups were calculated using a chi-square test. Since polymorphisms tested in complex diseases rarely account for a large amount of variance, characterized by very low *p*-values (*p* < 0.001), we decided to present the results without correction for multiple testing and using a *p*-value ≤ 0.05.

## Results

Daily energy and nutrient intakes measured by a 3-day food record are presented in Table 1. After the n-3 PUFA supplementation, saturated fat, carbohydrate and protein intakes were significantly different from the pre-supplementation period (p = 0.0008, p = 0.001 and p = 0.04 respectively). PUFA intakes (including fish oil capsules and food) were significantly higher after the supplementation (p = 0.003). Subjects were asked to limit their fish intake to no more than 2 servings/week (one serving of fish = 75 g) and the mean intake was 0.89 servings/week for the 6 weeks of the n-3 PUFA supplementation period. Based on these recommendations, subjects who had consumed the maximum quantity of fish permitted each week would have had an extra 0.43 g of EPA + DHA per day.

**Table 1:** Nutrients intakes before and after n-3 PUFA supplementation (n = 208)

**Nutrients**	**Pre-n-3 PUFA**^**1**^	**Post-n-3 PUFA**^**1**^	**p**^**2**^
Energy (kcal)	2272 ± 590	2186 ± 566	0.08
Total lipids (g)	84.5 ± 29.2	86.6 ± 29.8	0.47
MUFA (g)	30.8 ± 11.8	29.6 ± 12.4	0.27
PUFA (g)	15.2 ± 6.6	17.1 ± 6.9	0.003*
SFA (g)	29.0 ± 12.0	25.4 ± 10.4	0.0008*
Cholesterol (mg)	303.7 ± 147.4	297.3 ± 169.4	0.65
Carbohydrates (g)	286.7 ± 78.9	263.4 ± 77.7	0.001*
Protein (g)	97.8 ± 30.2	92.6 ± 29.6	0.04*
Alcohol (g)	3.2 ± 6.0	3.2 ± 6.1	0.99

*p < 0.05 ^1^Values are means ± SD, ^2^ANOVA for the differences between pre- and post-supplementation, adjusted for age, sex and BMI.

**Figure 2:**
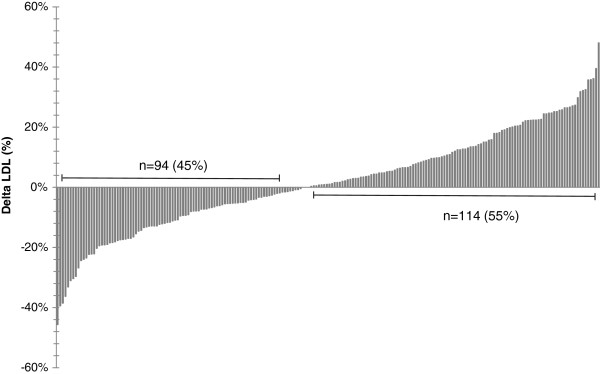
LDL-C variation by subject after the n-3 PUFA supplementation.

Biochemical and anthropometric characteristics of subjects before and after the n-3 PUFA supplementation are presented in Table 2. Differences in pre- versus post-supplementation were observed for plasma TG only. Subjects maintained their body weight, BMI and waist circumference stable during the protocol. Moreover, there were no change in plasma TC, HDL-C, ApoB as well as LDL particle size after the n-3 supplementation. LDL-C levels increased in 55% of subjects, as illustrated in Figure 2. The mean LDL-C variation was -0.35 ± 0.31 mmol/L (-12.4 ± 9.8%) in negative responders and +0.34 ± 0.26 mmol/L (+13.5 ± 10.6%) in positive responders (mean of all subjects +0.03 ± 0.45 mmol/L (+1.9 ± 16.5%)). The change in LDL-C levels was not different between men and women after the n-3 PUFA supplementation (data not shown).

**Table 2:** Subjects’ characteristics before and after the n-3 PUFA supplementation (n = 208; Men = 96 (46%) and Women = 112 (54%))

	**Pre**^**1**^	**Post**^**1**^	**p**^**2**^
Age (years)	30.8 ± 8.7		
Weight (kg)^4^	81.0 ± 13.7	81.2 ± 14.0	0.8
BMI (kg/m^2^)^3,4^	27.6 ± 3.4	27.7 ± 3.6	0.8
Waist circumference (cm)^4^	93.0 ± 10.0	93.0 ± 10.1	0.9
Total cholesterol (mmol/L)^5^	4.72 ± 0.88	4.69 ± 0.91	0.6
HDL (mmol/L)^5^	1.43 ± 0.35	1.46 ± 0.39	0.3
LDL (mmol/L)^5^	2.75 ± 0.82	2.77 ± 0.85	0.9
Triglycerides (mmol/L)^3,5^	1.19 ± 0.62	1.01 ± 0.51	0.0001*
Apolipoprotein B (g/L)^5^	0.83 ± 0.24	0.86 ± 0.23	0.2
LDL size (Å)^5^	254.06 ± 2.65	254.21 ± 2.79	0.6

*p < 0.05 ^1^Values are means ± SD, ^2^ANOVA for the differences before and after the supplementation, ^3^Values are log(10) transformed, ^4^Results adjusted for age and sex, ^5^Results adjusted for age, sex and BMI.

All SNPs tested were in HWE (Table 3) and are located in introns. The MIXED procedure for repeated measures was used to test for potential interactions between SNPs of *MGLL* and the n-3 PUFA supplementation on blood lipids. Genotype, supplementation and genotype*supplementation interaction were included in 18 models (one for each SNP) adjusted for age, sex and BMI. Plasma TG, total cholesterol and HDL-C levels did not show any genotype or genotype*supplementation interactions effects (data not shown). Results for LDL-C levels and LDL particle size are shown in Tables 4 and 5, respectively. Only the SNPs with significant values for either genotype or genotype*supplementation interaction effects are shown. For LDL-C, neither supplementation effects nor genotype effects were observed for any of the SNPs studied. However, six SNPs (rs782440, rs6776142, rs555183, rs782444, rs6787155, rs1466571) were significantly interacting with n-3 PUFA supplementation to modulate plasma LDL-C levels. Furthermore, four SNPs were associated with LDL particle size (rs782440, rs13076593, rs549662, rs541855). Three genotype*supplementation interaction effects were observed for SNPs of *MGLL* on LDL particle size (rs782440, rs9877819, rs13076543). The results remained unchanged after further adjustments for either energy, PUFA, SFA, protein or carbohydrate intakes at baseline (data not shown). After further adjustments for changes in carbohydrate and protein intake during the supplementation, results remained the same except for the association between rs78244 and plasma LDL-C levels that was no longer significant afte adjustment for changes in protein intake.

**Table 3:** **Selected SNPs of the *MGLL *** **gene**

**dbSNP**	**Sequence**	**MAF**^**1–2**^	**Genotype/Frequency**		
rs782440	ACCAGC[C/T]TGTGCA	0.50	C/C (n = 53)	C/T (n = 105)	T/T (n = 52)
			0.25	0.50	0.25
rs16826716	GTTTCC[C/T]GTCATG	0.09	C/C (n = 172)	C/T (n = 38)	T/T (n = 0)
			0.82	0.18	0.00
rs6776142	CTGTCA[C/T]GCAGAG	0.33	C/C (n = 91)	C/T (n = 98)	T/T (n = 21)
			0.43	0.47	0.10
rs9877819	ATACAC[A/G]AGGTGT	0.17	G/G (n = 145)	A/G (n = 59)	A/A (n = 6)
			0.69	0.28	0.03
rs555183	AGAGGC[A/G]CCATCA	0.43	A/A (n = 70)	A/G (n = 101)	G/G (n = 39)
			0.33	0.48	0.19
rs6780384	CCTGGG[G/T]AGAAAG	0.10	G/G (n = 168)	G/T (n = 40)	T/T (n = 2)
			0.80	0.19	0.01
rs13076593	TCCAAG[C/G]TAGTAA	0.12	C/C (n = 163)	C/G (n = 44)	G/G (n = 3)
			0.78	0.21	0.01
rs605188	TCTGGG[C/T]GTCTGG	0.42	C/C (n = 72)	C/T (n = 98)	T/T (n = 40)
			0.34	0.47	0.19
rs6765071	CATGAC[C/T]ACGTTC	0.23	C/C (n = 126)	C/T (n = 73)	T/T (n = 44)
			0.60	0.35	0.21
rs782444	GGGCCA[C/T]AGGCAG	0.43	C/C (n = 72)	C/T (n = 94)	T/T (n = 44)
			0.34	0.45	0.21
rs549662	TGCGGT[A/G]AGTGTG	0.17	A/A (n = 145)	A/G (n = 59)	G/G (n = 6)
			0.69	0.28	0.03
rs3773155	CCCCCA[A/G]TCGCAC	0.12	A/A (n = 161)	A/G (n = 46)	G/G (n = 3)
			0.77	0.22	0.01
rs541855	GTGAGA[C/T]GAAAGG	0.18	C/C (n = 139)	C/T (n = 65)	T/T (n = 6)
			0.66	0.31	0.03
rs6439081	ATGCCA[C/T]CACATG	0.24	T/T (n = 121)	C/T (n = 78)	C/C (n = 11)
			0.58	0.37	0.05
rs6439082	CATCCC[C/T]GATCAG	0.15	C/C (n = 154)	C/T (n = 49)	T/T (n = 7)
			0.73	0.23	0.03
rs6787155	CGGACA[A/C]GGTTTA	0.22	A/A (n = 130)	A/C (n = 66)	C/C (n = 14)
			0.62	0.31	0.07
rs1466571	CCAGGT[A/G]AAGAGA	0.33	G/G (n = 91)	A/G (n = 98)	A/A (n = 21)
			0.43	0.47	0.10
rs893294	TGAGGA[A/T]GGATGG	0.34	A/A (n = 93)	A/T (n = 91)	T/T (n = 26)
			0.44	0.43	0.12

^1^Minor allele frequency.

^2^Allelic and genotypic frequencies were obtained using the ALLELE procedure in SAS Genetics V9.3.

**Table 4:** Genotype, supplementation and genotype*supplementation interaction effects on LDL-C levels after an n-3 PUFA supplementation

**SNP**	**Genotype**	**β (interaction term)**	**P Genotype**	**P Supplementation**	P Genotype* Supplementation
rs782440	C/C	-0.05	0.8	0.1	0.01*
	C/T	-0.03			
	T/T	0			
rs6776142	C/C	0.11	0.6	0.08	0.008*
	C/T	0.05			
	T/T	0			
rs555183	A/A	-0.03	0.9	0.1	0.047*
	A/G	-0.14			
	G/G	0			
rs782444	C/C	-0.01	0.8	0.4	0.048*
	C/T	-0.10			
	T/T	0			
rs6787155	A/A	-0.17	0.7	0.8	0.02*
	A/C	-0.25			
	C/C	0			
rs1466571	A/A	-0.08	0.8	0.1	0.02*
	A/G	-0.07			
	G/G	0			

*p*-values are derived from log10-transformed data; All results were adjusted for age, sex and BMI; The MIXED procedure in SAS V9.2 was used to test for interaction effects.

*p<0.05.

**Table 5:** Genotype, supplementation and genotype*supplementation interaction effects on LDL particle size after an n-3 PUFA supplementation

**SNP**	**Genotype**	**β (interaction term)**	**P Genotype**	**P Supplementation**	P Genotype* Supplementation
rs782440	C/C	-1.57	0.03*	0.2	0.047*
	C/T	-1.09			
	T/T	0			
rs9877819	A/A + A/G	0	0.2	0.6	0.002*
	G/G	0.92			
rs13076593	C/C	1.50	0.006*	0.85	0.02*
	C/G + G/G	0			
rs549662	A/A	-1.13	0.02*	0.02*	0.07
	A/G + G/G	0			
rs3773155	A/A	-0.34	0.8	0.02*	0.1
	A/G + G/G	0			
rs541855	C/C	1.19	0.006*	0.2	0.2
	C/T + T/T	0			

*p*-values are derived from log10-transformed data; All results were adjusted for age, sex and BMI; The MIXED procedure in SAS V9.2 was used to test for interaction effects.

*p<0.05.

Subsequently, genotype frequencies were compared between positive responders and negative responders as defined on the basis of their plasma LDL-C response to the supplementation. Genotype frequency differences were observed for rs782440, rs555183, rs6780384, rs782444 and rs6787155 (Table 6). For rs782440 and rs555183, homozygotes for the rare allele were more likely to be negative responders. For rs6780384, rs782444 and rs6787155, homozygotes for the major allele were more likely to be negative responders. Then, we divided the subjects by their LDL particle size variation. Only one SNP (rs549662) showed a significant genotype frequency difference as the minor allele homozygotes were more likely to increase their LDL particle size (data not shown).

**Table 6:** Differences in genotype frequencies of five SNPs according to the subject’s plasma LDL-C response to an n-3 PUFA supplementation

**SNP**	**Homozygotes (wild-type)**		**Heterogygotes**		**Homozygotes (rare)**		**p**^**1**^
	**P-Responders**	**N-responders**	**P-Responders**	**N-responders**	**P-Responders**	**N-responders**	
rs782440	23 (11%)	30 (14%)	56 (27%)	47 (23%)	15 (7%)	37 (18%)	0.01
rs555183	27 (13%)	42 (20%)	54 (26%)	46 (22%)	13 (6%)	26 (13%)	0.04
rs6780384	68 (33%)	99 (47%)	26 (13%)	13 (6%)	0 (0%)	2 (1%)	0.006
rs782444	23 (11%)	48 (23%)	48 (23%)	45 (22%)	23 (11%)	21 (10%)	0.03
rs6787155	49 (24%)	80 (38%)	38 (18%)	27 (13%)	7 (3%)	7 (3%)	0.02

^1^chi-square test in SAS 9.2.

P (positive) responders versus N (negative) responders.

## Discussion

In the present study, we tested the effects of *MGLL* gene polymorphisms on plasma LDL-C and LDL particle size following an n-3 PUFA supplementation. The *MGLL* gene has been previously shown to be differentially expressed in peripheral blood mononuclear cells (PBMCs) between subjects responders and non-responders defined on the basis of the plasma TG levels variation in pre- versus post- n-3 PUFA supplementation. 18 SNPs were genotyped to cover 100% of the common genetic variations. To our knowledge, this is the first study to examine the influence of *MGLL* gene variations on LDL-C levels and LDL particle size in relation to an n-3 PUFA supplementation.

Near half of the subjects increased their LDL-C levels (55%) and the plasma LDL-C variation ranged from -1.7 mmol/L to +1.0 mmol/L, which shows the large inter-individual variability of the LDL-C response to the n-3 PUFA supplementation observed in this cohort. In response to dietary interventions, many studies also found an important inter-individual variability of the plasma LDL-C. In studies with the National Cholesterol Education Panel step 2 diets, Schaefer et al. found a plasma LDL-C variation ranging between +13% and -55% between participants [41]. A review also showed that LDL-C varied from +17% to +46% in hypertriglyceridemic subjects receiving 4 g/day of n-3 PUFA [42]. We also found that the LDL particle size was not statistically different in pre- versus post-supplementation period in our study, while many studies found an increase in the LDL particle size following an n-3 PUFA supplementation, as described earlier. It has been described earlier that the LDL particle size is inversely related to plasma TG concentrations [43]. In a study by Griffin et al., altering the n-6/n-3 ratio by giving PUFA enriched diet to subjects has been found to decrease TG levels as well as the proportion of small, dense LDL particles [17]. In addition, Kelley et al. showed a decrease in TG levels and a increase in LDL particle size following a DHA supplementation in hypertriglyceridemic men [21]. The TG levels in our cohort decreased by 11%, as reported previously [44], compared to a mean of 25% decreased following 3–4 g/day n-PUFA in normolipemic subjects in a meta-analysis [45]. This may potentially explain the lack of difference in LDL particle size after the n-3 PUFA supplementation. Dietary intakes in relation to LDL particle size variation have been studied by Faghihnia et al. reporting that a low-fat, high-carbohydrate diet reduces LDL particle size [46]. However, in the present study, results remained unchanged after further adjustment for dietary intakes. The lack of difference in effect of the n-3 PUFA supplementation on LDL particle size may also be attributable to the large inter-variability observed in the plasma TG levels in our study cohort, as previously reported [47].

We verified the independent effects of supplementation and genotype as well as the supplementation*genotype interaction effects on LDL-C and LDL particle size. No supplementation or genotype effects were observed for LDL-C. However, six SNPs (rs782440, rs6776142, rs555183, rs782444, rs6787155, rs1466571) of *MGLL* showed significant supplementation*genotype effects on LDL-C, suggesting that these variants may modulate the LDL-C response to an n-3 PUFA supplementation. Four SNPs of *MGLL* (rs782440, rs13076593, rs549662, rs541855) were associated with LDL particle size. The T/T carriers for the rs782440 SNP, the C/C for rs13076593, the A/G + G/G genotypes combined for rs549662 and the C/C genotype for rs541855 had a beta value of 0 or higher for LDL particle size comparatively to the other genotype groups. Three SNPs showed a supplementation*genotype interaction (rs782440, rs9877819, rs13076543). The rs782440 SNP is present in all these statistical associations. To our knowledge, genetic variants of *MGLL* have only been studied once in the literature in relation to metabolic outcomes in humans. An association study of Harismendy et al. showed that in a population of obese individuals as well as non-obese controls, three intervals of rare variants in the *MGLL* gene sequence are associated with BMI (promoter, intron 2 and intron 3) [48].

The MGLL enzyme is known to hydrolyse 2-AG, which is one of the central components of the endocannabinoid signaling network [31]. The activation of the cannabinoid receptors by 2-AG have an impact on energy homeostasis by stimulating appetite, promoting lipid storage and reducing energy expenditure. They have also been associated with metabolic changes associated with obesity and metabolic syndrome [49]. Circulating 2-AG levels in the plasma have been correlated positively with BMI and waist girth, as well as with plasma TG levels [50], while another study found that 2-AG levels were increased by 52% in obese women [51]. These higher levels of 2-AG in obese individuals may result of a reduced enzymatic degradation by the MGLL enzyme. Although we did not find any associations between *MGLL* SNPs and BMI or waist circumference, these measures have been strongly associated to plasma lipid levels [52] and LDL particle features [53]. However, a study with MGLL-KO mice showed that despite an elevation of 2-AG levels, animals did not have enhanced lipid storage, increased appetite or decreased energy expenditure [54].

The allele frequency disparities observed between the different genotype groups and the positive responder/negative responder status for LDL-C variation suggests that individuals with different genotypes for these SNPs may respond differently to an n-3 PUFA supplementation.

## Conclusion

In conclusion, we did not found any effect of the n-3 PUFA supplementation on plasma LDL-C concentrations and LDL particle size after six weeks, but we observed a large inter-individual variability in the LDL-C response to the supplementation. Also, this study suggests that SNPs within the *MGLL* gene modulate LDL-C and particle size during an n-3 PUFA supplementation. Specific genotypes also seem to influence the variation in LDL-C levels following the supplementation. However, further investigation will be needed to fully explain the complex interactions underlying these associations.

### Consent

Written informed consent was obtained from all subjects for the publication of this report.

## Competing interests

Benoit Lamarche has received funding from Atrium Innovations for the study of commercially available EPA and DHA supplements in the context of metabolic syndrome. Other authors declare no competing interests.

## Authors’ contributions

CO participated in the meeting of participants, conducted genotyping, performed statistical analysis and wrote the paper; IR, SL and MCV designed research; BL contributed to the measure of the LDL particle size; PC was responsible for the medical follow-up; CO and MCV have primary responsibility for final content. All authors read and approved the final manuscript.
